# The Properties of Different Healing Agents Considering the Micro-Self-Healing Process of Asphalt with Encapsulations

**DOI:** 10.3390/ma14010016

**Published:** 2020-12-22

**Authors:** Benan Shu, Min Zhou, Tengyu Yang, Yongling Li, Yunlong Ma, Kai Liu, Shiwen Bao, Diego Maria Barbieri, Shaopeng Wu

**Affiliations:** 1 Foshan Transportation Science and Technology Co., Ltd., Foshan 528000, China; shuba0411@126.com (M.Z.); mly11082510@126.com (T.Y.); lyling19941209@163.com (Y.L.); myl11082510@163.com (Y.M.); 2 School of Automobile and Traffic Engineering, Hefei University of Technology, Hefei 230009, China; liukai@hfut.edu.cn; 3 Chizhou Re-Art New Material Co., Ltd., Chizhou 247000, China; baosw@whut.edu.cn; 4 Department of Civil and Environmental Engineering, Norwegian University of Science and Technology, NO-7491 Trondheim, Norway; diego.barbieri@ntnu.no; 5 State Key Laboratory of Silicate Materials for Architectures, Wuhan University of Technology, Wuhan 430070, China

**Keywords:** healing agent, self-healing, asphalt, molecular dynamics simulation, encapsulation

## Abstract

Improving the self-healing performance of asphalt by employing encapsulation technology is a topic of wide interest. This study investigated the performance of sunflower oil, engine oil, and waste cooking oil based on the microhealing mechanism of asphalt with compartmented polymeric fiber. Capillary flow, contact angle, Brookfield viscosity, bar thin layer chromatography, and fatigue–recovery–fatigue tests were conducted to characterize the capillary flow capacity, wetting ability, viscosity reduction ability, suitability of components, and performance restoration ability of the different kinds of healing agents. The diffusion process of sunflower oil in asphalt was simulated using molecular dynamics. The results showed that sunflower oil exhibited the best capillary flow capacity, viscosity reduction ability, and the fastest wetting rate in asphalt. Engine oil exhibited the largest wetting work and the best recovery performance related to fatigue. The diffusion process of sunflower oil in asphalt could be divided into two stages. Two major factors (aging and higher temperature) increased the diffusion rate of sunflower oil in asphalt. The comprehensive analysis showed that sunflower oil was the most suitable to be encapsulated to improve the self-healing performance of asphalt.

## 1. Introduction

In recent years, different technologies have been formulated to improve the self-healing abilities of asphalt to prolong its service life, such as the addition of nanoparticles [1,2], electromagnetic/microwave induction [3,4,5], and encapsulations [6,7,8]. The encapsulations can be ruptured by the development of cracks, which thus trigger the crack-healing process [9]. Many types of encapsulations have been synthesized, such as melamine formaldehyde (MMF) microcapsules [10], melamine urea formaldehyde resin (MUF) microcapsules [11], and calcium alginate capsules/fiber [12,13]. The materials forming the encapsulations need to withstand the temperature and the mechanical stress related to the mixing and compaction process used on an asphalt mixture.

Traditionally, the encapsulated healing agents are classified according to two types based on the different healing mechanisms, namely, chemical reactions and non-chemical reactions. Liu et al. [14] used water-based epoxy resin as a healing agent. After the rupture of encapsulation generated by the crack propagation, the water-based epoxy resin flowed out and a chemical cross-linking reaction occurred between the healing agent and curing agent, where cracks could be filled with the products of the reaction. Dimethylphenol (DMP) employed as the healing agent was encapsulated and examined in Chung’s work [15]. When cracks are generated inside asphalt, a polymerization reaction occurred and dimethylphenol would be converted into polyphenylene oxide under the catalysis of oxygen in the air and metals in the asphalt, such as vanadium, and the cracks were gradually filled by the agent.

The healing agents that belong to the non-chemical reactions category mainly includes all kinds of light oil. This kind of healing agent is mainly composed of a high content of saturates and aromatics. The effectiveness of this kind of healing agent is to be found in the small molecular dimensions and the subsequent lubrication effect. In addition, both the large π bond in the aromatics, which can improve the colloidal stability and adhesion of asphalt, and the saturates, which are able to reduce the viscosity of asphalt, can account for the self-healing performance of asphalt [16]. Alvaro Garcia et al. [17] prepared a cement/epoxy resin capsule that encapsulates sunflower oil. The experimental results showed that the healing agents could soften the asphalt near cracks to improve the fluidity of asphalt and accelerate the healing of cracks inside asphalt. Computed tomography (CT) results showed that the crack area of an asphalt mixture with capsules was significantly smaller than that of an asphalt mixture without capsules. Norambuena Contreras et al. [18] fabricated Ca-alginate capsules containing sunflower oil and it was found that the strength recovery of the asphalt mixture could be improved by approximately 30% after the addition of encapsulated sunflower oil. Shirzad et al. [19] conducted a comparative study on the rheological recovery properties of aged asphalt by using sunflower oil and a proprietary chemical stabilizer. It was found that the former one could restore the aged asphalt more effectively. Therefore, sunflower oil was employed as the core material used in microcapsules to improve the self-healing properties of asphalt. Sun et al. [20] investigated light oil contained in microcapsules where the core materials used had a high content of aromatics as the core material of the microcapsules. The healing agent was composed of 28.4% saturates and 70.9% aromatics. The addition of encapsulated light oil could double the fatigue life of an asphalt mixture. The tensile stress recovery test showed that the strength recovery rate of asphalt containing 5% microcapsules increased by 30.35%. Su et al. [21] synthesized microcapsules encapsulating waste cooking oil. The fluorescence microscope showed that the healing agent could easily permeate into asphalt, soften and improve the self-healing property of asphalt.

Researchers have accomplished thorough investigations focusing on improving the self-healing performance of asphalt using an encapsulated healing agent, but there is a general lack of knowledge when it comes to comparing the performance of the different healing agents at a microscale level. A kind of compartmented fiber-containing healing agent was synthesized by the authors’ group to improve the self-healing performance of asphalt [22]. The healing agent was encapsulated in calcium alginate fiber in the form of droplets. The content of the encapsulated healing agent was about 43%. The diameter and wall thickness of the compartments were about 400 and 50 μm, respectively.

According to the previous work of the authors [23], the microhealing process can be divided into the following stages: the rupture of the fiber compartments due to crack development, the release of the healing agent, filling of the crack thanks to capillary action, rapid diffusion of the healing agent into the asphalt, a sharp decrease in asphalt viscosity, and subsequent disappearance of the crack. The main factors that affect the efficiency of the considered healing agent include capillary flow, wetting, viscosity, chemical compositions, diffusion, and performance restoration. Based on these premises, the abovementioned properties belonging to sunflower oil, engine oil, and waste cooking oil were comparatively evaluated in this work based on the microhealing mechanism of compartmented fiber in this work.

## 2. Materials and Methods

### 2.1. Materials

**Figure 1:**
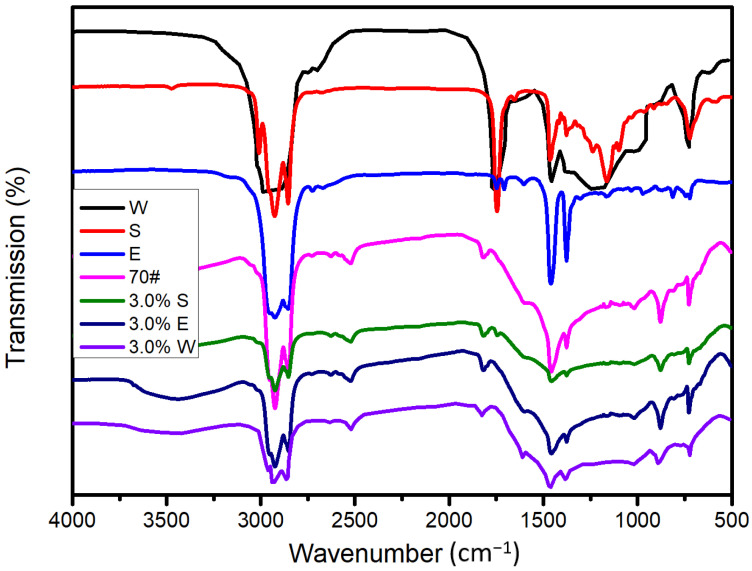
Fourier-transform infrared spectroscopy (FTIR) spectra of the asphalt and studied healing agents (sunflower oil (S), engine oil (E), waste cooking oil (W)).

70# asphalt (asphalt with a 60/80 penetration grade) was bought from KOCH Bitumen Co. Ltd (Wuhan, China). The penetration (0.1 mm) and softening points were 68.7 and 48.5 °C, respectively. Sunflower oil (S) was supplied by Arowana Group Co. Ltd (Wuhan, China). Engine oil (E) was bought from AMER Technology Co. Ltd (Wuhan, China). Waste cooking oil (W) was supplied by a local restaurant (Wuhan, China). The FTIR results of the three kinds of healing agents are shown in Figure 1.

### 2.2. Methods

Fourier-transform infrared spectroscopy (FTIR) tests were performed (Nexus, Thermo Nicolet, New York, NY, USA) with wavenumbers ranging from 500 to 4000 cm^−1^. The three kinds of healing agents were added to the asphalt (3% refers to the total bitumen mass, where the codes were 3.0% S, 3.0% E, and 3.0% W, respectively).

**Figure 2:**
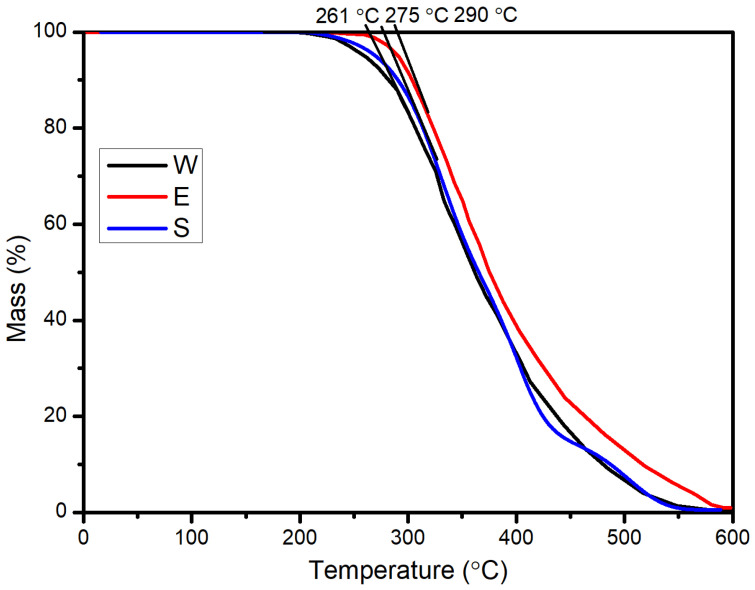
Thermal stability of the three kinds of healing agents (sunflower oil (S), engine oil (E), waste cooking oil (W)).

It is necessary to study whether a chemical reaction takes place after the addition of the healing agent to the asphalt. Figure 2 shows that W had a characteristic infrared peak similar to S, and both had a strong characteristic peak at 1747 cm^−1^. Comparing the peaks of S and E, it was found that the main difference was the intensity of the four characteristic peaks. The intensity of the characteristic peaks at 1747 cm^−1^ and 1163 cm^−1^ in S were both much larger than those of E, while the peak intensities of E at 1464 cm^−1^ and 1377 cm^−1^ were both much larger than those of S. The peak at 1747 cm^−1^ was due to the stretching vibration peak of C=O, and the peak at 1163 cm^−1^ was due to the stretching vibration peak of C–O. The result indicated that S and W had more oxygen functional groups, and the unsaturation of the two kinds of healing agents was relatively strong. The peaks at 1464 cm^−1^ and 1377 cm^−1^ were due to the bending vibration peak of methylene, which indicates that the carbon chain of E was longer. In addition, after the addition of the healing agent, the characteristic peaks of the asphalt and the healing agent could both be detected, and there was no new characteristic peak. This shows that the mixing of S, E, and W with the asphalt was physical blending.

Thermogravimetric analysis (TGA) tests were conducted (STA449c/3/G, NETZSCH, Berlin, Germany) for temperatures ranging from 30 to 600 °C in atmospheric air. The heating rate was 10 °C/min throughout the test. The TGA investigated the thermal stability of the three kinds of healing agents was tested, where the results are displayed in Figure 2. It was found that the initial decomposition temperatures of S, E, and W were 275 °C, 290 °C, and 261 °C, respectively, which indicates that the three kinds of healing agents had excellent thermal stability and met the temperature requirement (about 160 °C) related to asphalt mixing and compaction.

Capillaries with a length of 100 mm and an inner diameter of 200 μm were used to test the capillary flow of the different healing agents. The three kinds of healing agents were each filled in glass dishes that had a diameter of 100 mm at 15 °C. During the test process, one end of the capillary was inserted vertically into the healing agent. The whole experiment lasted for 1 min and the capillary remained stationary. One minute later, the height of the healing agent in the capillary was measured with vernier calipers.

**Figure 3:**
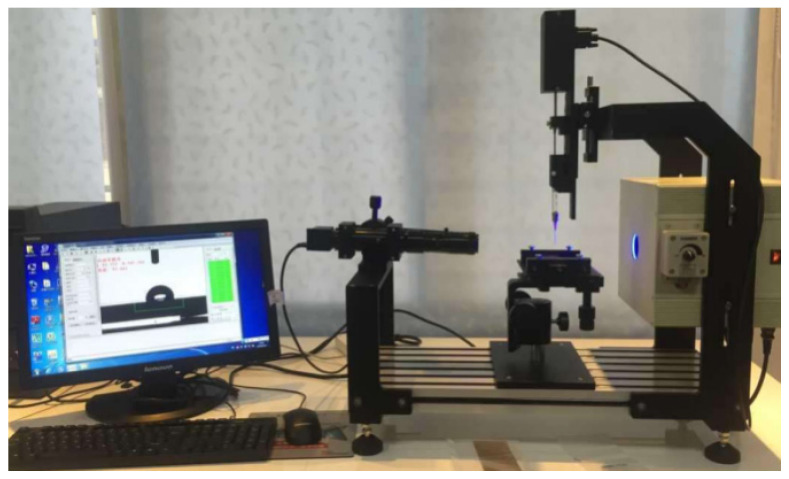
Contact angle tester, which consists of three parts: an automatic injection system, a charge-coupled device (CCD) camera, and an automatic image analysis system.

The surface tension of the three kinds of healing agents was tested using a DCAT 21 instrument (Dataphysics, Filderstadt, Germany), where the machine is shown in Figure 3. The test temperature was kept at 15 °C. The contact angles of the three kinds of healing agents were measured on a JY-82B machine (Kruss DSA, Hamburg, Germany).

**Figure 4:**
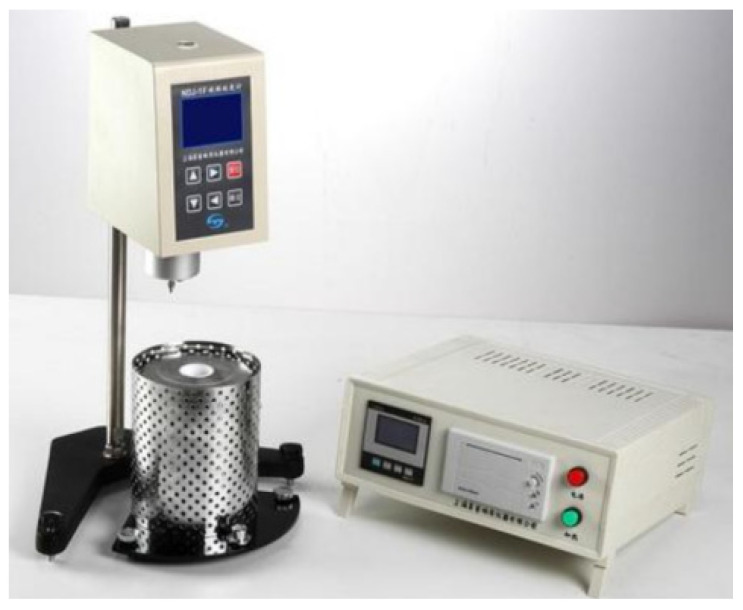
Brookfield rotational viscometer, which consists of two parts: a temperature control system and a shear rate control system.

A Brookfield rotational viscometer (NDJ-1, Manufacturer, Beijing, China) was used to test the viscosity of the asphalt at 60 °C (the machine is shown in Figure 4). The test process was operated according to the specification JTG E20-2011 (Beijing, China).

The relative contents of saturates, aromatics, resins, and asphaltenes of the asphalt were assessed by employing the thin-layer chromatography detection method (TLC-FID). The experiment was performed on an Iatroscan MK-6 machine (IATRON, Tokyo, Japan). For this experiment, the asphalt was first dissolved in dichloromethane solution. In order to isolate the four components, N-heptane, toluene/ethanol (55:45), and toluene/heptane (80:20) were adopted sequentially. Organic ions of different kinds of components were generated via the calcination of a hydrogen flame. FID detected the specific current intensity generated by the organic ions. The larger the current intensity, the more content there was of that specific component of the asphalt.

**Figure 5:**
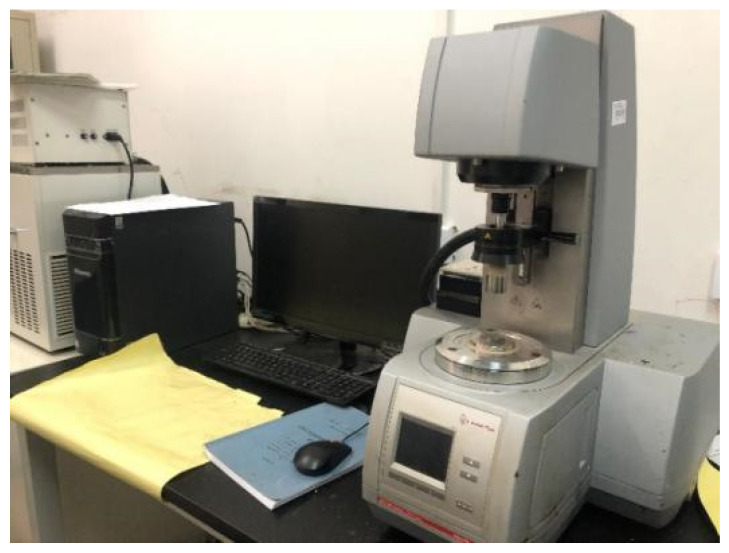
Dynamic shear rheometer (DSR) equipment consists of a temperature control system, a stress and strain control system, and an automatic data analysis system.

A dynamic shear rheometer (DSR) model MCR101 (Anton Par, Canberra, Australia) was used to study the fatigue recovery of the asphalt with the different kinds of healing agents (the machine is shown in Figure 5).

A rotor with a diameter of 8 mm was used. The strain and frequency were kept at 3% and 10 Hz, respectively. The test temperature and healing temperature were controlled at 15 and 30 °C, respectively. The modulus recovery and fatigue cycles recovery were thus obtained. The three indexes—HI_1_, HI_2_, and HI_3_—evaluated the fatigue cycles recovery ability of the asphalt, which are defined as follows [24]:

(1) $${\mathrm{HI}}_{1}=\frac{G_{healing}^{*}-G_{terminal}^{*}}{G_{initial}^{*}-G_{terminal}^{*}}\times 100\%$$

(2) $${\mathrm{HI}}_{2}=\frac{G_{healing}^{*}-G_{terminal}^{*}}{G_{initial}^{*}-G_{terminal}^{*}}\times \frac{N_{after}}{N_{before}}\times 100\%$$

(3) $${\mathrm{HI}}_{3}=\frac{E_{after}}{E_{before}}\times 100\%,\;E=\int_{1}^{n}E_{i},\;E_{i}=\delta_{i}\times \epsilon_{i}$$

where $G_{healing}^{*}$ is the modulus of the asphalt sample after the healing period, $G_{initial}^{*}$ is the initial modulus of the asphalt sample, $G_{terminal}^{*}$ is the modulus of 70% of $G_{initial}^{*}$, $N_{after}$ is the fatigue loading cycles of the asphalt after the healing period when the $G_{healing}^{*}$ is reduced to $G_{terminal}^{*}$, $N_{before}$ is the initial fatigue loading cycles when the initial modulus is reduced to $G_{terminal}^{*}$, $E_{after}$ is the strain energy of the asphalt after the healing period, $E_{before}$ is the initial strain energy of the asphalt, and $\delta_{i}$ and $\epsilon_{i}$ are the strain and stress at the *i*th loading cycle, respectively. Table 1 shows the codes used in the manuscript and their interpretations.

## 3. Results and Discussion

### 3.1. Capillary Flow Property

The formula of the capillary height h is shown as follows:

(4) $$h=\frac{2\gamma \cos\theta }{\rho gr}$$

where *γ* is the liquid surface tension of the liquid, *θ* is the contact angle between the liquid and solid surface, ρ is the density of the liquid, g is the acceleration due to gravity, and *r* is the radius of the capillary.

**Figure 6:**
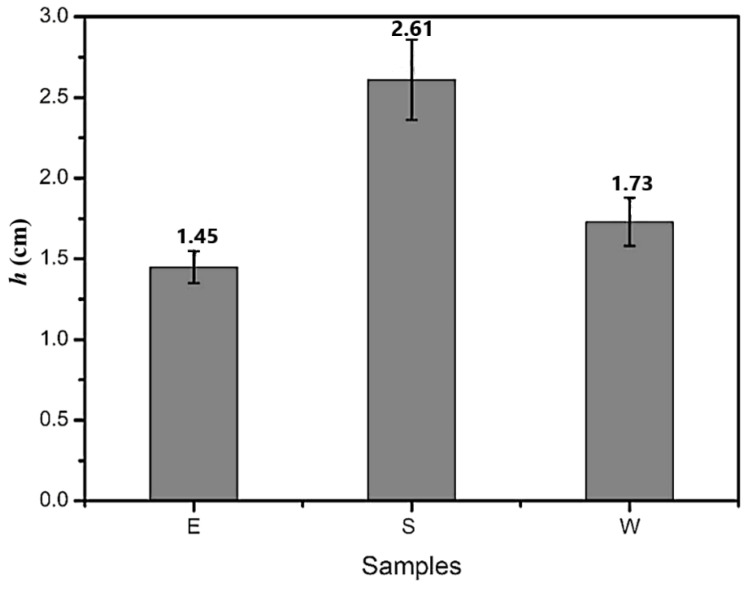
Capillary flow capacity of the three kinds of healing agents (sunflower oil (S), engine oil (E), waste cooking oil (W)).

During the asphalt self-healing process, the internal microcrack is similar to the capillary and a larger capillary height of the healing agent indicates that more healing agents can be filled in the crack; it is more conducive to improving the self-healing properties of asphalt. Figure 6 shows the capillary flow capacity of the three healing agents at 15 °C. It was found that S had the largest capillary height, which was 2.61 cm, followed by W with a capillary height of 1.73 cm. E had the smallest capillary height, which was 1.45 cm. After the crack formation, the healing agent encapsulated in the fiber is released and filled the crack under capillary action. When the healing agent has better capillary flow capacity, the crack area is filled by more healing agent, which is conducive for healing cracks more efficiently and quickly.

### 3.2. Wetting Property

The surface tension of a liquid is directly related to the capillary flowability of the liquid. Infiltration means that the solid could be adhered to by a liquid. Adhesion can be divided into three types: spreading, sticking, and wetting. The contact angle of spreading is about zero. The contact angle of adhesion is less than 180°. The contact angle of wetting is less than 90°. The contact angles of the three kinds of healing agents were found to be less than 90° using the lying drop method, which indicates that asphalt could be wetted by the three kinds of healing agents. The change in free energy in this process is as follows:

(5) $$-\Delta G_{i}=G_{sg}-G_{sl}=W_{i}$$

where $G_{sg}$ is the free energy of the solid surface, $G_{sl}$ is the free energy of the “solid–liquid” interface, and $W_{i}$ is the wetting work. The larger the wetting work is, the easier it is for the healing agent to adhere and spread on the asphalt surface. In addition, the wetting process can be described using Young’s equation, which is shown in the following formula:

(6) $$G_{l}\cos\theta =G_{s}-G_{sl}$$

where $G_{l}$ is the free energy of the liquid surface. The wetting work obtained from the combination of the above two formulas is as follows:

(7) $$W_{i}=\gamma_{lg}\cos\theta$$

where $\gamma_{lg}$ is the surface tension of the liquid. The spreading speed can be expressed in terms of the wetting speed, which is shown as follows:

(8) $$\frac{d\cos\theta^{\prime }}{dt}=\frac{f_{l}}{\eta d}(\cos\theta -\cos\theta^{\prime })$$

where $\cos\theta^{\prime }$ is the contact angle of the healing agent on the asphalt surface at time *t*, $f_{l}$ is the surface tension of the healing agent, *η* is the viscosity of the healing agent, *d* is the fluid width, *θ* is the contact angle of the healing agent on the asphalt surface in a steady state; when *t* → 0, the contact angle can be approximated as 180°, *d* can be approximated as 1, and the wetting speed is as follows:

(9) $$\vartheta =\frac{f_{l}}{\eta }\left(\cos\theta +1\right)$$

This formula was used to evaluate the wetting rate of the healing agents on the asphalt surface.

The viscosity, surface tension, and contact angles of the three kinds of healing agents were tested, and the results are shown in Table 2.

**Figure 7:**
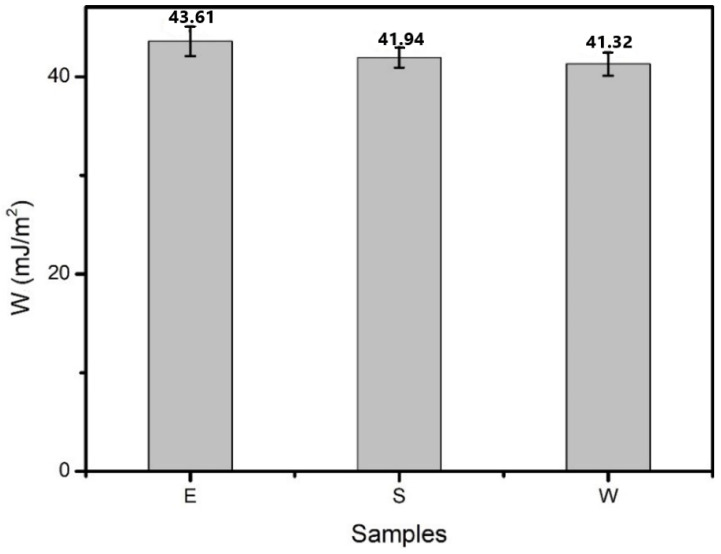
Wetting work of the three kinds of healing agents.

**Figure 8:**
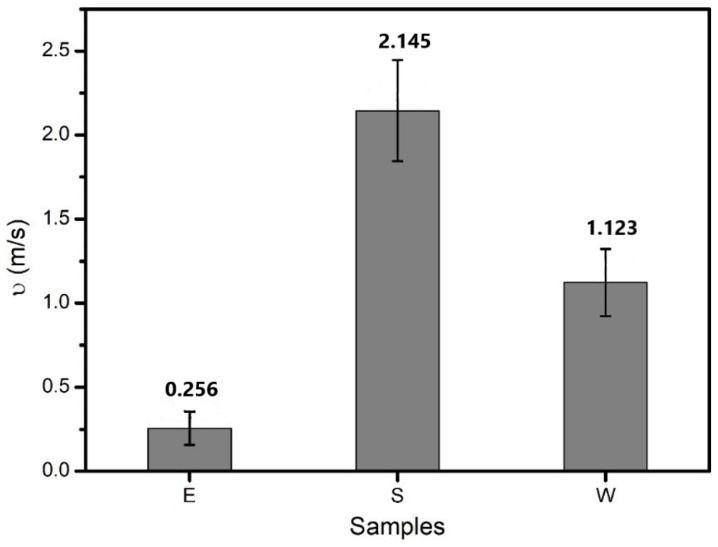
Wetting rate of the three kinds of healing agents.

Figure 7 and Figure 8 display the wetting work and wetting speed of the three kinds of healing agents. It can be seen that the wetting work of E was slightly larger than that of S and W. The wetting speed results revealed that S had the largest value (2.145 m/s), followed by W (1.123 m/s). The wetting speed of E was the smallest. Cracks in asphalt are filled up with a healing agent due to capillary action. The larger the wetting work and wetting speed are, the easier it is for the healing agent to adhere to the asphalt surface, which is conducive for playing the role of a healing agent and improving the self-healing performance of asphalt. The results showed that E could more easily adhere to the asphalt surface, which was attributed to the smallest contact angle. S had the largest wetting rate, which means that compared with E and W, S could penetrate the cracks more within the same time.

### 3.3. Viscosity Reduction Ability

**Figure 9:**
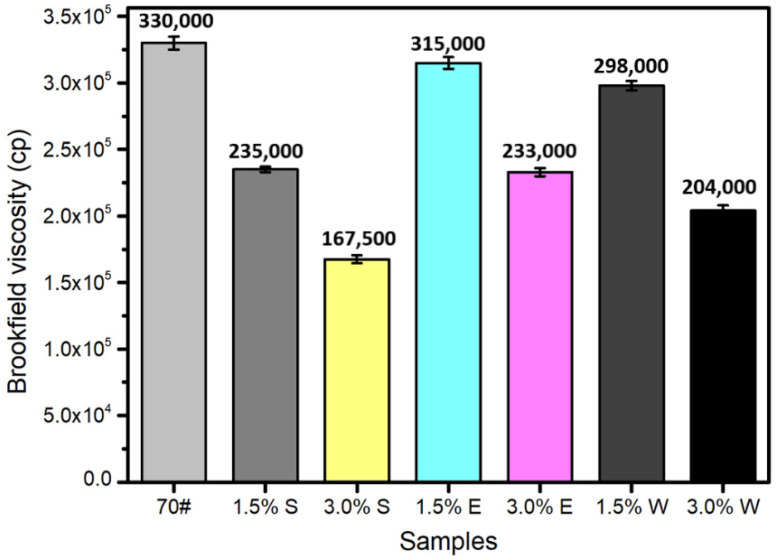
Viscosity of the asphalt with different kinds of healing agents.

Viscosity has a direct effect on the self-healing performance of asphalt [25]. The smaller the viscosity, the better the flow properties of asphalt. Cracks can thus be filled and healed by asphalt more quickly. Overall, the viscosity of the asphalt was significantly reduced by the presence of healing agents (Figure 9). S had the best viscosity reduction effect, followed by W and E, where the outcome related to using 3.0% E was equivalent to that of using 1.5% S. The viscosity of the asphalt containing 1.5% S and the asphalt containing 3% S were about 2.3 × 10^5^ cp and 1.68 × 10^5^ cp, respectively, which were approximately 30% and 49% smaller than the virgin asphalt. From the analysis of the viscosity reduction effect, S had the best performance when it came to improving the flow ability of the asphalt.

**Figure 10:**
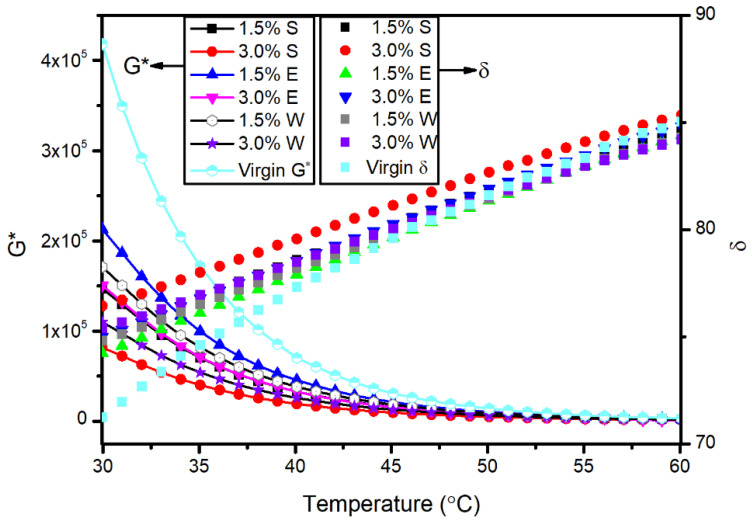
Complex modulus and phase angle of the asphalt with different kinds of healing agents.

The complex modulus and phase angle of the asphalt with different kinds of healing agents versus temperature are shown in Figure 10. The complex modulus describes the hardness degree of the asphalt, and the phase angle describes the relative proportion of the viscous and elastic components in the asphalt. The results show that the changes in the complex modulus and the phase angle were similar to the changes in the viscosity of the asphalt with different kinds of healing agents. The three kinds of healing agents softened the asphalt, where the sunflower oil had the best performance.

### 3.4. Four Fractions Results

**Figure 11:**
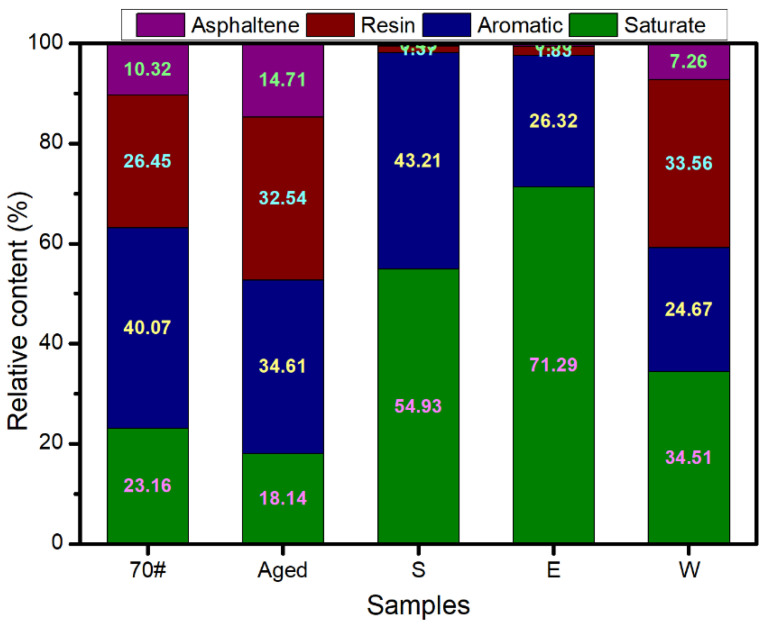
Relative content of the four components of the virgin asphalt (70#), aging asphalt, and the three kinds of healing agents.

The relative contents of saturates, aromatics, resin, and asphaltene of the three kinds of healing agents were obtained through the four-component experiment (SARA). The experimental results are shown in Figure 11. After the aging of the 70# virgin asphalt, the relative contents of the saturates and aromatics were both reduced, and the contents of the resin and asphaltene were increased. Therefore, the asphalt became hard and brittle after being aged, and it was easy for cracks to generate inside the asphalt under the action of an external environment. It was necessary to supplement the saturates and aromatics, which can not only soften the asphalt and improve the self-healing ability of the asphalt but also supplement the lightweight components of the asphalt, restructure the colloidal characteristic, and restore the performance of the asphalt. It can be seen from Figure 11 that the relative content of the saturate and aromatic components in S, E, and W were 98.14%, 97.61%, and 59.18%, respectively. Therefore, the performances of S and E were better than W in terms of the relative content of light components.

### 3.5. Performance Restoration

**Figure 12:**
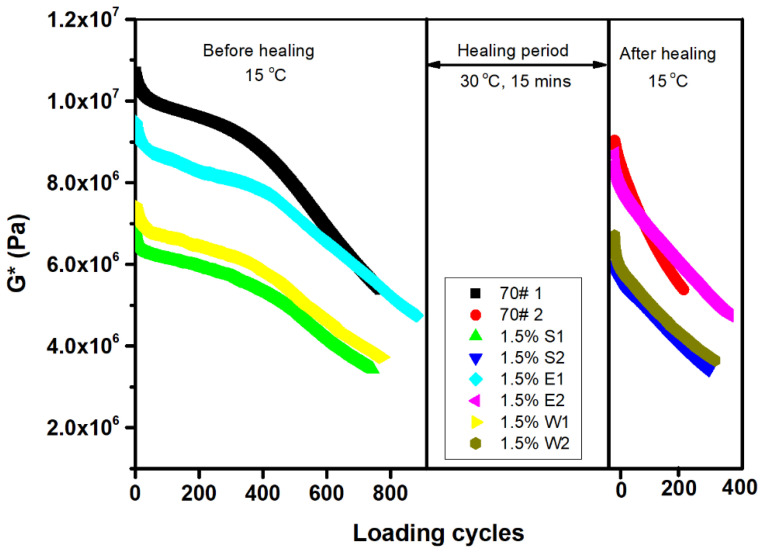
Fatigue–healing–fatigue test results of the asphalt containing the different healing agents.

Figure 12 shows the results of the effect of S, E, and W on the fatigue performance recovery of the asphalt. It was found that S had the largest reduction effect on the initial modulus of the asphalt. In contrast, E had the lowest reduction effect on the initial modulus of the asphalt. It is worth noting that the modulus reduction rate of the asphalts containing the different healing agents was much slower than that of the 70# virgin asphalt under the action of fatigue loading. Specifically, the modulus reduction rate of the asphalt containing E was the smallest. From Table 3, when the initial modulus was reduced to half, the asphalt with E had the largest number of fatigue loading cycles, which were significantly higher than that of the virgin asphalt. After 15 min of self-healing at 30 °C, when the modulus was reduced to $G_{terminal}^{*}$, all three kinds of healing agents significantly improved the number of fatigue loading cycles of the asphalt, indicating that all three kinds of healing agents played a role in improving the performance restoration of the asphalt. According to the self-healing indexes of the three kinds of asphalt shown in Table 3, the self-healing rates of the three indexes were different, which indicates that different results were obtained when using different indexes to evaluate the self-healing performance of the asphalt. Compared with the indexes of the three kinds of asphalt, the three indexes of the asphalt with 1.5% E were all the largest. Compared with S and W, E had a better effect on the performance restoration of the asphalt.

**Figure 13:**
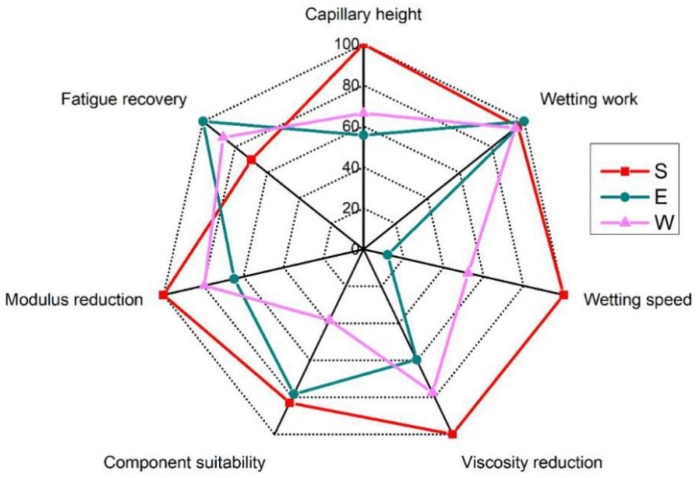
Radar chart of the performance of the three healing agents based on the microhealing mechanisms of compartmented fiber.

In order to more intuitively compare the performances of the three kinds of healing agents in terms of all factors affecting the self-healing performance of the asphalt, a radar chart was drawn, which is shown in Figure 13. The best performance of the three healing agents in each property index was set to 100, and the value of the other two healing agents was the ratio to the best performance. It was found that the capillary height, wetting speed, viscosity complex, and modulus reduction of S were all 100. The wetting work and fatigue recovery of E were set to 100 since these performances were the best. S had the largest area; therefore, S is the recommended healing agent for the synthesis of encapsulation to improve the self-healing performance of the asphalt. After the adherence of S on the asphalt, the diffusion process of S in the asphalt needed to be simulated and studied in this work.

### 3.6. Molecular Dynamics Simulation

#### 3.6.1. Establishment of the Molecular Model

**Figure 14:**
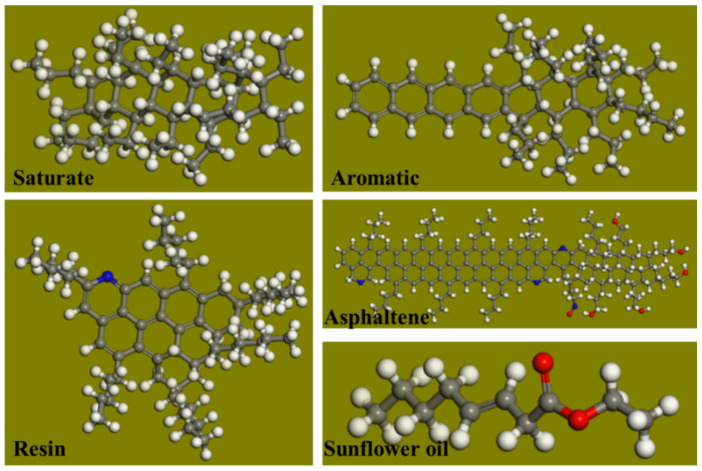
The molecular models of saturates, aromatics, resins, asphaltenes, and sunflower oil.

An asphalt molecular model can be built using a three-component method or a four-component method [26,27,28]. Zhou et al. [29,30] point out that the four-component model is suitable for the study of the physical, chemical, and rheological properties of asphalt. Therefore, the four-component model was selected in this work to build the asphalt molecular model (the asphalt molecular model and sunflower oil model are shown in Figure 14; for information regarding how the molecular structure of the asphalt was constructed, refer to [31]).

**Figure 15:**
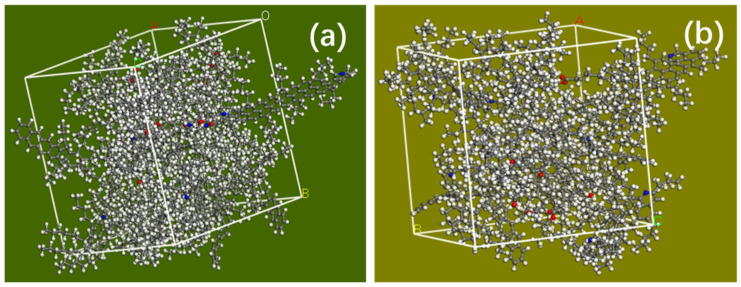
Molecular models of virgin asphalt (**a**) and aged asphalt (**b**) containing 3% sunflower oil.

The molecular models of the virgin and aging asphalt mixed with 3% S were constructed, and the result is shown in Figure 15.

**Figure 16:**
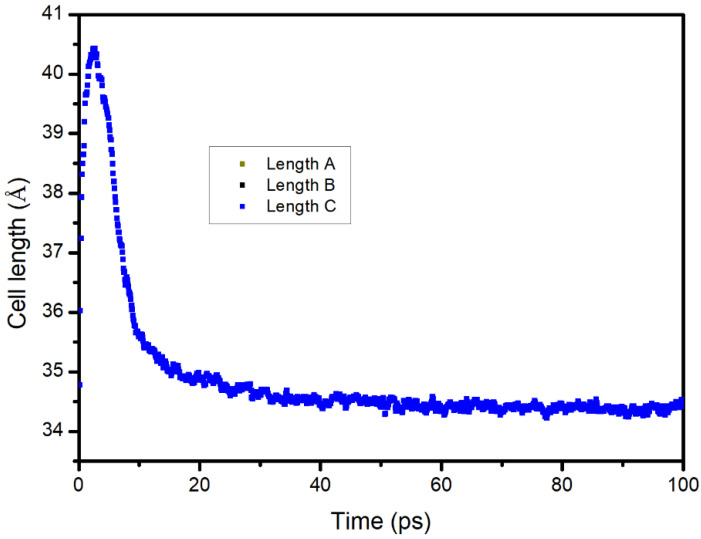
Change in the system volume with time during the diffusion process of sunflower oil in the asphalt.

**Figure 17:**
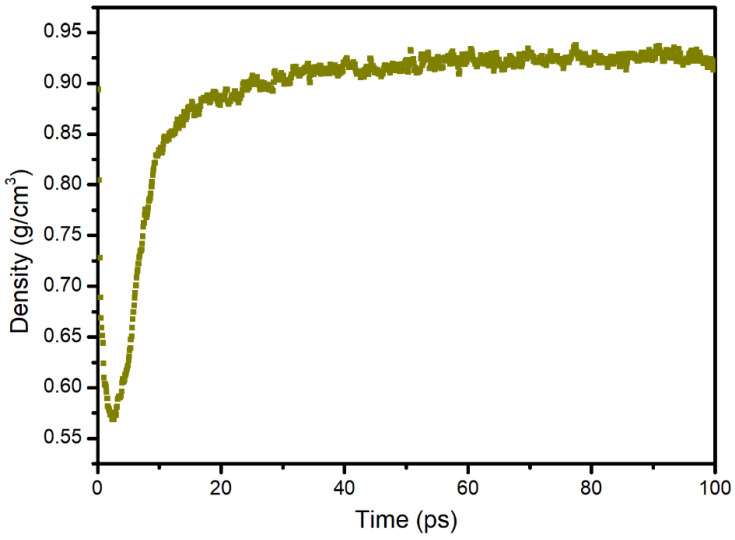
Change in the system density with time during the diffusion process of sunflower oil in the asphalt.

The changes in the volume and density of the asphalt during the diffusion process were calculated, and the results are shown in Figure 16 and Figure 17. It was found that the volume decreased rapidly with increased diffusion time and then remained stable. The density increased rapidly in the early stage and remained stable in the later stage. The reason for this was that in the early diffusion stage, S rapidly filled the pores in the asphalt; therefore, the volume decreased rapidly, and the density increased rapidly. Then, the diffusion was in equilibrium; therefore, the density and volume were constant. Based on the result, the simulation time was determined to be 100 ps (10^−10^ s).

#### 3.6.2. Effect of Temperature

Based on statistical theory, the mean square displacement (MSD) can reflect the motion law of a large number of atoms. The formula is as follows:

(10) $$\mathrm{MSD}\left(t\right)=\langle {\left|\gamma \left(t\right)-\gamma \left(0\right)\right|}^{2}\rangle$$

where 〈〉 is the average value of all atoms in the system, and *γ*(*t*) is the position of atoms at time t in the system.

Based on the fluctuation dissipation theory of non-equilibrium statistical thermodynamics, the diffusion coefficient D can be calculated according to the parameter MSD. The calculation formula is as follows:

(11) $$D=\underset{t\to \infty }{\lim}\frac{1}{6t}\times \mathrm{MSD}\left(t\right)$$

The diffusion coefficient reflects the motion intensity of a molecule/atom in the material. The higher the diffusion coefficient is, the higher the diffusion rate of the molecule/atom.

**Figure 18:**
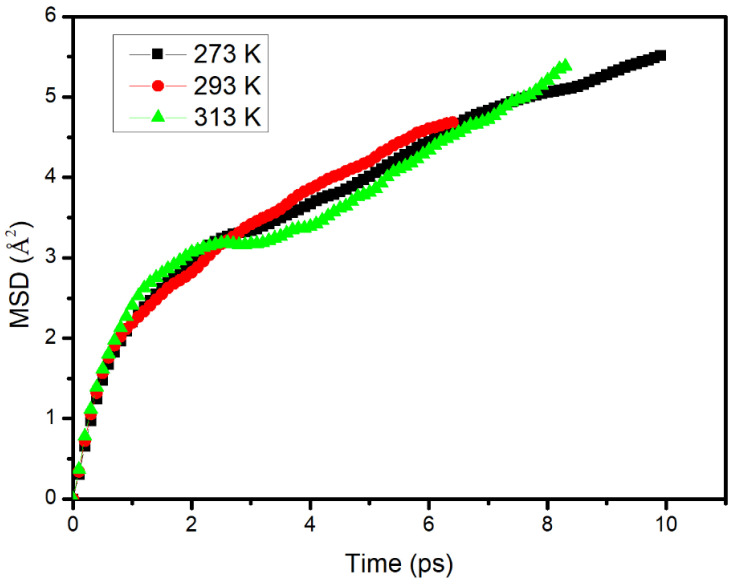
Change in the mean square displacement over time of sunflower oil in the asphalt.

The change in the MSD over time describes the law of the movement of molecules of the diffusion substance from contacting with the substrate to diffusing stably in the substrate. According to the above formula for the diffusion coefficient, the diffusion coefficient is 1/6*t* of MSD when the diffusion time tends to infinity. The change in MSD of S with time in the asphalt under different temperatures was simulated and the result is shown in Figure 18. It can be seen that the diffusion process of S in the asphalt could be divided into two stages. The first stage was between 0–3 ps. In this stage, diffusion molecules rapidly contacted and filled the pores in the matrix. Thus, the MSD increased rapidly with time. In the second stage, the diffusion of molecules in the matrix was in equilibrium; therefore, MSD increased linearly with the diffusion time.

**Figure 19:**
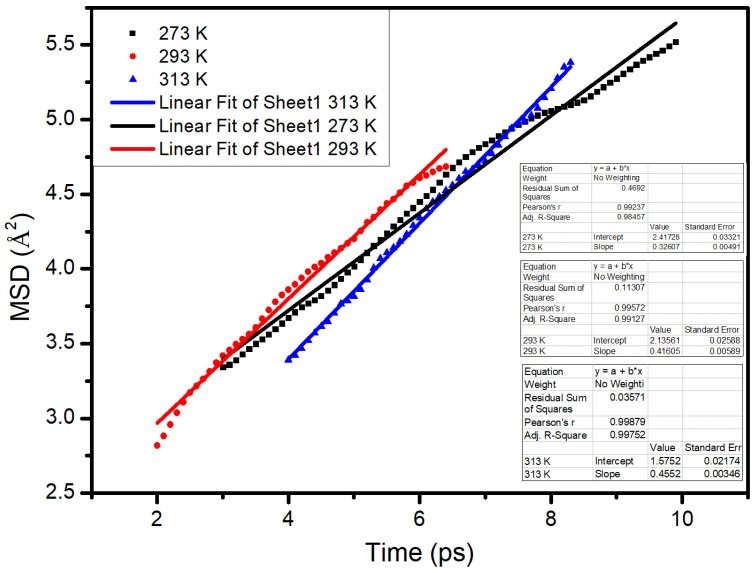
Fitting results of the second stage over time.

The diffusion coefficient was calculated based on the linear fitting of the second stage, where the result is shown in Figure 19. It can be found that the fitting slopes of the second stage at 0 °C, 20 °C, and 40 °C were 0.3261, 0.4161, and 0.4552, respectively. The diffusion coefficients of S in the asphalt at 0 °C, 20 °C, and 40 °C were 5.43 × 10^−10^ m^2^/s, 6.934 × 10^−10^ m^2^/s, and 7.587 × 10^−10^ m^2^/s, respectively. With the increase of temperature, the diffusion coefficient of S in the asphalt gradually increased. Molecular motion needs energy. The higher the temperature, the greater the molecular energy. The asphalt molecules had better diffusion ability.

#### 3.6.3. Effect of the Asphalt Aging

**Figure 20:**
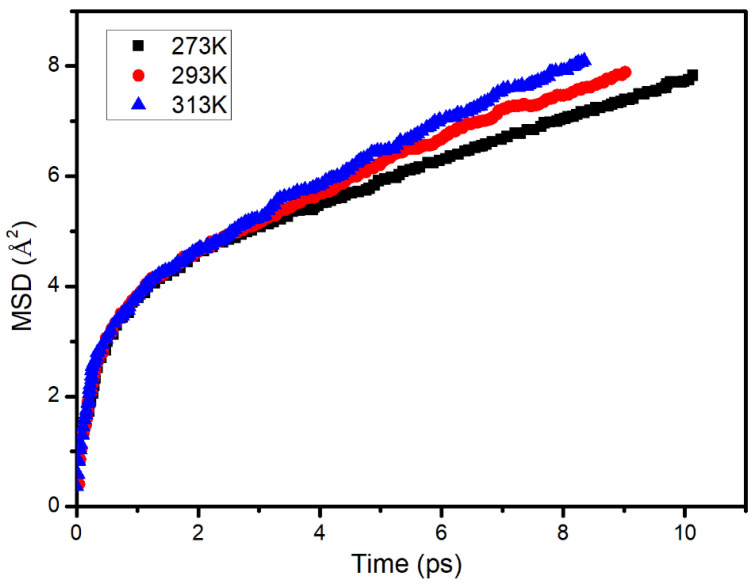
Change in the mean square displacement over time of sunflower oil in the aged asphalt.

The change in the MSD with time in the aged asphalt is shown in Figure 20. It was found that the diffusion law of S in the aged asphalt was consistent with that of the virgin asphalt. When the temperature was 0 °C, 20 °C, and 40 °C, the diffusion rate of S in the aged asphalt was 6.269 × 10^−10^ m^2^/s, 7.256 × 10^−10^ m^2^/s, and 8.676 × 10^−10^ m^2^/s, respectively.

The difference between the diffusion rates of S in the virgin and aged asphalt is shown in Table 4. It was found that the diffusion rate of S in the aged asphalt was greater than that in the virgin asphalt. After the aging process, the number of pores in the asphalt increased, which was conducive to the diffusion of S. In addition, the content of heavy components (such as asphaltene) also increased, and the content of light components decreased [32,33,34]; therefore, the concentration of light components in the aged asphalt was lower than that of the virgin asphalt. Under the effect of the increased porosity and the concentration difference, S exhibited a faster diffusion in the aged asphalt.

## 4. Conclusions

In this work, the performance of three kinds of healing agents (sunflower oil, engine oil, and waste cooking oil) was thoroughly evaluated by considering the micro-self-healing mechanism of encapsulation in asphalt. The diffusion process of sunflower oil in asphalt was simulated using molecular dynamics. Based on the above results and analysis, the following conclusions were drawn:

1. Sunflower oil showed the best capillary flow capacity, viscosity reduction ability, and the largest infiltration rate. The capillary length and wetting rate at 15 °C were 2.61 cm and 2.145 m/s, respectively. The viscosity of the asphalt containing 3.0% S was reduced by 49%.
2. Engine oil exhibited the best wetting and fatigue recovery ability. The wetting work at 15 °C was 43.61 mJ/m^2^. The healing indexes HI_1_, HI_2_, and HI_3_ were 83.8%, 34.2%, and 36.1%, respectively.
3. The diffusion process of sunflower oil in the asphalt could be divided into two stages, namely, a rapid contact diffusion stage and a stable diffusion stage. The increase in temperature and the aging of the asphalt increased the diffusion rate of sunflower oil.
4. Comprehensive analysis shows that sunflower oil had the best performance regarding improving the asphalt self-healing; therefore, sunflower oil is the most suitable healing agent of those tested for the fabrication of encapsulation to improve the self-healing performance of asphalt.

## Figures and Tables

**Table 1:** Codes used in the manuscript and their interpretations.

Codes	Interpretation	Codes	Interpretation
1.5% S, 3.0% S	Asphalt samples with 1.5 wt.% and 3 wt.% sunflower oil, respectively	1.5% S1, 1.5% S2	Asphalt samples with 1.5 wt.% sunflower oil before and after healing, respectively, in the fatigue–healing–fatigue test
1.5% E, 3.0% E	Asphalt samples with 1.5 wt% and 3 wt.% engine oil, respectively	1.5% E1, 1.5% E2	Asphalt samples with 1.5 wt.% engine oil before and after healing, respectively, in the fatigue–healing–fatigue test
1.5% W, 3.0% W	Asphalt samples with 1.5 wt.% and 3 wt.% waste cooking oil, respectively	1.5% W1, 1.5% W2	Asphalt samples with 1.5 wt.% waste cooking oil before and after healing, respectively, in the fatigue–healing–fatigue test
Three groups of parallel samples tests were carried out for the performance experiments on the asphalt			

**Table 2:** Surface tension and contact angle of the three kinds of healing agents.

Healing Agent	Viscosity (cp)	Surface Tension (mN/m)	Contact Angle (°)/cosθ
E	367	50.40	30.1/0.8652
S	45	54.56	39.8/0.7687
W	83	51.87	37.2/0.7965

**Table 3:** Performance restoration of the asphalt with different kinds of healing agents.

Samples	$G_{initial}^{*}\;(\mathrm{MPa})$	$G_{terminal}^{*}\;(\mathrm{MPa})$	$G_{healing}^{*}\;(\mathrm{MPa})$	$N_{before}\;(\mathrm{cycles})$	$N_{after}\;(\mathrm{cycles})$	$HI_{1}\;(\%)$	$HI_{2}\;(\%)$	$HI_{3}\;(\%)$
70#	10.7	5.38	9.03	768	217	67.9	19.2	24.9
1.5% S	6.86	3.43	6.01	744	297	75.3	30.0	35.1
1.5% E	9.48	4.74	8.72	881	360	83.8	34.2	36.1
1.5% W	7.45	3.73	6.70	775	315	79.6	32.3	35.3

**Table 4:** Diffusion rate of sunflower oil in the virgin and aged asphalt at different temperatures.

Samples	Temperature (°C)	*D* (10^−10^ m^2^/s)	R^2^
70# virgin + 3% S	0	5.439	0.9846
	20	6.934	0.9912
	40	7.587	0.9975
70# aging + 3% S	0	6.269	0.9981
	20	7.256	0.9894
	40	8.676	0.9962

## Data Availability

Data sharing not applicable. No new data were created or analyzed in this study. Data sharing is not applicable to this article.
